# Dynamics of Water Clusters Confined in Ionic Liquid at an Elevated Pressure

**DOI:** 10.1021/acs.jpclett.4c00356

**Published:** 2024-03-18

**Authors:** Amith Kumar Murali, Marian Paluch, Riccardo Casalini, Alyna Lange, Andreas Taubert, Zaneta Wojnarowska

**Affiliations:** † Institute of Physics, University of Silesia in Katowice, 75 Pulku Piechoty 1A, 41-500 Chorzow, Poland; ‡ Chemistry Division, Naval Research Laboratory, 4555 Overlook Avenue Southwest, Washington, D.C. 20375, United States; § Institute of Chemistry, University of Potsdam, Karl-Liebknecht-Straße 24-25, 14469 Potsdam-Golm, Germany

## Abstract

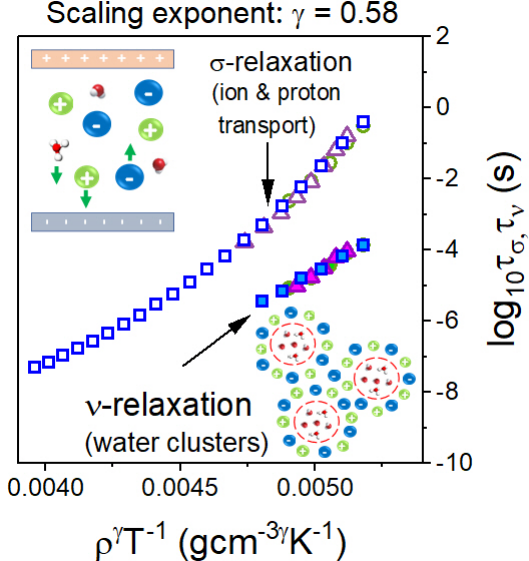

Over the years, numerous experimental and theoretical efforts have been dedicated to investigating the mysteries of water and determining its new unexplored physical properties. Despite this, high-pressure studies of water and aqueous mixtures close to the glass transition still represent an unknown area of research. Herein, we address a fundamental issue: the validity of the density scaling concept for fast water dynamics. For this purpose, we performed ambient and high-pressure dielectric measurements of a supercooled equimolar aqueous mixture of an acidic ionic liquid. All isothermal and isobaric relaxation data describing the time scale of charge transport (τ_σ_) and fast dynamics within the water clusters (τ_ν_) reveal visual evidence of a liquid–glass transition. Furthermore, both relaxation processes satisfy the ρ^γ^/*T* scaling concept with a single exponent γ = 0.58. Thus, the scaling exponent is a state-point-independent parameter for the dynamics of water clusters confined in ionic liquid investigated in the pressure range up to 300 MPa.

Water, a seemingly simple molecule composed of two hydrogen atoms and one oxygen, possesses remarkable properties that have intrigued scientists across diverse fields of study for decades.^1^ Density anomalies, high specific heat capacity, or high surface tension make water important in our natural world, fascinating for various scientific disciplines, and relevant to many applications.^2^ The fact that water defies conventional expectations and behaves in ways that set it apart from other simple liquids arises from its small size combined with high polarity and, thus, capability to form an extensive hydrogen-bonding network that varies dynamically with the temperature and pressure.^3−5^ Consequently, various thermodynamic and dynamic properties of water go through a minimum when plotted as a function of the temperature, pressure, or density.

The mysterious properties of water become even more pronounced in a supercooled state below 273 K.^6^ For example, two forms of water, high-density (HD) and low-density (LD) water, separated by a first-order liquid–liquid phase transition and differing in relaxation behavior (fragile versus strong), were found at various *T*–*P* conditions.^7−11^ At the same time, one of the essential properties of amorphous water, that is, the glass transition temperature, remains unclear.^12−14^ It has been found that glassy water, also called amorphous ice, exists when the temperature drops below 130 K under ambient conditions. However, later on, from the measurement of multiple hyperquenched glasses, it was argued that the correct *T*_g_ should be closer to 165 K.^15^ All of these controversies arise from the fact that water is challenging to vitrify and easily crystallize in the so-called no man’s land region, i.e., between about 150 and 235 K.^16^ Accordingly, several approaches have been explored in the literature to hinder the homogeneous nucleation of water and investigate its relaxation dynamics. One includes distortion in the hydrogen-bonding network via confinement in nanoscale structures.^17−19^ Alternatively, studies of aqueous mixtures serve to explore relaxation dynamics in the temperature range below 235 K.^20−22^ In the latter approach, water contributes to the global structural relaxation by reducing the liquid–glass transition temperature.^23^ Furthermore, local motions of H_2_O molecules are a source of secondary ν relaxation observed in dielectric relaxation spectra,^24,25^ with the activation energy of the ν mode being similar to that found for the faster process detected under confinement.^26^

A valuable advantage of impedance spectroscopy is that molecular dynamics of glass-forming liquids can be investigated at an elevated pressure.^27^ Nevertheless, the dielectric response of water or aqueous mixtures in the vicinity of the liquid–glass transition is relatively rarely investigated under pressure,^28^ while dynamic behavior across the whole temperature–pressure–volume (*T*–*P*–*V*) thermodynamic space in terms of density scaling ρ^γ^/*T*, with the γ exponent reflecting the repulsive part of the effective short-range intermolecular potential,^29,30^ is still unexplored. Consequently, it has never been verified whether the two processes characterizing the dynamics of aqueous mixtures near *T*_g_, structural α and local ν relaxation, are controlled by the same potential. Another unanswered question is whether the water in clusters satisfies the density scaling or follows the behavior of bulky water at *T* > 298 K that breaks the scaling rule.^31^

To address these issues, we have prepared an equimolar homogeneous mixture of water and protic ionic liquid (IL) 1-methyl-3-(3-sulfobutyl)imidazolium *para*-toluenesulfonate ([BMIm-SO_3_H][pTS]) that vitrifies at 236 K and can be classified as a good glass former. Furthermore, as a result of the sulfonate/sulfonic acid group located in the chemical structure of ions, an extensive hydrogen-bonding network and fast proton transport between ionic species and water molecules is observed in this system, which makes it promising for fuel cell applications.,^32^ The isothermal and isobaric dielectric measurements combined with ρ(*T*,*P*) data show that, in the pressure range limited to 350 MPa, the ν and σ relaxations, reflecting, respectively, dynamics of water clusters and charge transport of the whole mixture, satisfy the density scaling with the same γ exponent being lower than unity.

The IL–H_2_O mixture examined herein was chosen as a model system to investigate water dynamics under ambient and high-pressure conditions. The ionic component of the mixture, [BMIm-SO_3_H][pTS], with the liquid–glass transition temperature at 260 K, can be classified as good glass-forming liquids without any crystallization tendency even on a slow cooling/heating rate (1 K/min) (see Figure 1). Furthermore, it is relatively hygroscopic as a result of its acidic nature. Therefore, even after extensive drying, IL still contained 0.5% of water. Because water substantially decreases *T*_g_ of [BMIm-SO_3_H][pTS], the mixture with 6.3 wt % H_2_O (that corresponds to a mole fraction of water *x*_H_2_O_ = 0.58) was chosen for further studies. This means that approximately one molecule of water per ion pair exists in the examined system. As presented in Figure 1, *T*_g_ of the chosen IL–H_2_O mixture drops by 24 K compared to pure IL, which falls in the temperature range suitable for ambient and high-pressure dielectric measurements. An additional advantage of the chosen composition is a stable water concentration over time.

**Figure 1:**
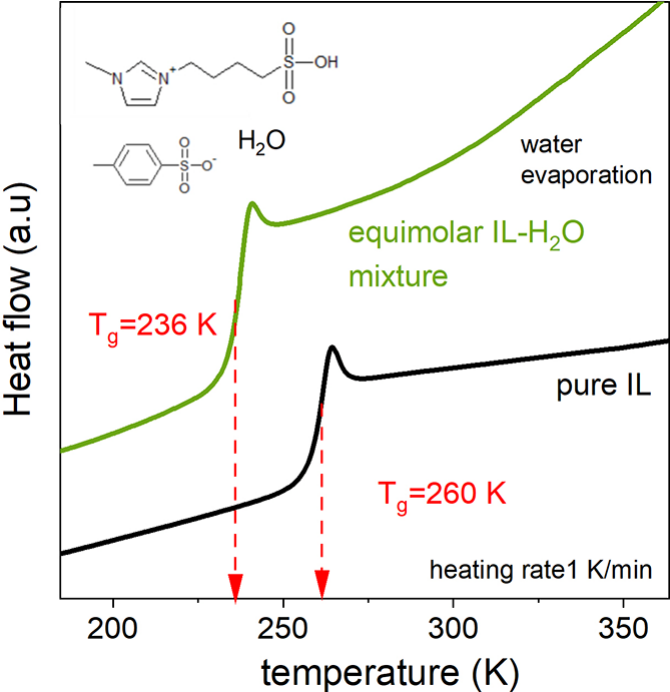
Differential scanning calorimetry (DSC) traces (*endo* up) of an equimolar mixture of water and [BMIm-SO_3_H][pTS] IL and pure unhydrous IL.

The representative dielectric spectra on IL–H_2_O composition measured over a broad range of frequency (*f* = 10^–2^–10^6^ Hz) and temperature (*T* = 155–287 K) by means of a Novocontrol Alpha analyzer (see the Supporting Information for experimental details) are presented in Figure 2a. Furthermore, the dried IL (containing 0.5 wt % water) was also examined as a reference (Figure 2b). The modulus formalism M*(*f*) = *M*′ + *iM*″ has been chosen to visualize the experimental data.^33^ This is because it is the only representation that allows for the characterization of ion-containing systems in glassy and supercooled liquid states.^34,35^ As shown in panels a and b of Figure 2, three relaxation processes are visible in the experimental frequency window of IL–H_2_O and dried IL. Above *T*_g_, the imaginary part of the electric modulus *M*″(*f*) takes the form of a well-resolved σ process, ascribed to the translational motions of ionic species. As the temperature decreases, the σ peak maximum shifts toward lower frequencies, which reflects a substantial slowing of charge carriers, and disappears from the dielectric window below the calorimetric *T*_g_. At the same time, a few kelvins above *T*_g_, the second relaxation appears in the dielectric spectra. Because its amplitude is markedly lower in dried IL (with remaining 0.5 wt % water) compared to an equimolar IL–H_2_O composition, we ascribed this process to the water dynamics and called it ν relaxation. Below *T*_g_, when the ion transport is negligible, the ν mode is still well-resolved in the frequency dispersion of *M*″; however, it reveals a much lower temperature sensitivity. Deep in the glassy state, the third relaxation, denoted as the β process, characterizes the dynamics of dried IL and the IL–H_2_O mixture. In contrast to ν relaxation, the β mode is more detectable in dried IL. This suggests some intramolecular motions within the cation, e.g., rotation of the sulfobutyl substituent, as a source of this process. To characterize thoroughly the water dynamics and charge transport in the examined IL–H_2_O mixture and its ionic component, we analyzed the relaxation spectra of both systems through the Havriliak–Negami function (see the Supporting Information) and determined precisely the characteristic relaxation times of all modes, σ, β, and ν.

**Figure 2:**
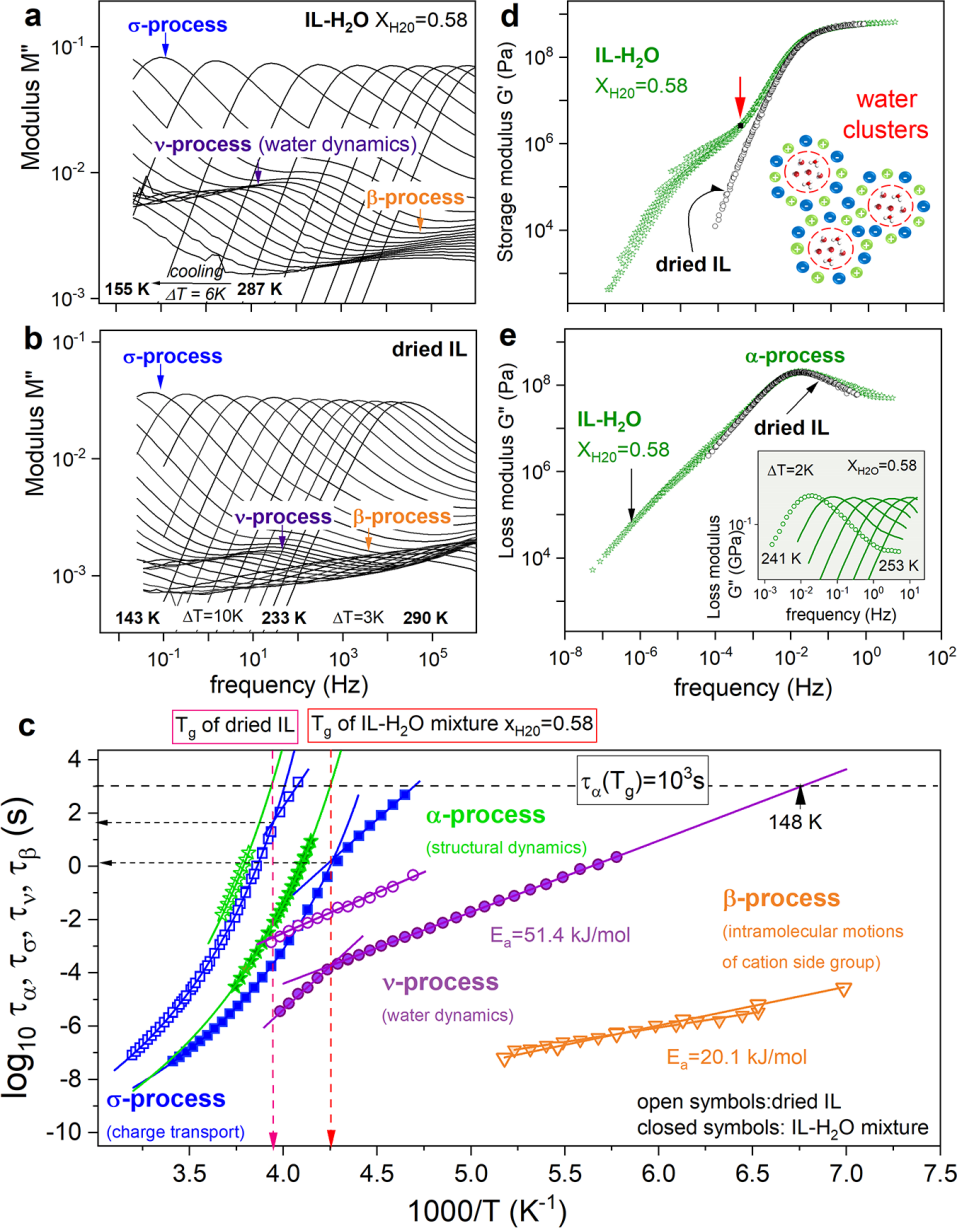
Representative dielectric spectra of (a) IL–H_2_O mixture and (b) dried [BMIm-SO_3_H][pTS] containing 0.5 wt % water presented in modulus formalism. (c) Relaxation map of IL–H_2_O and dried IL. Solid lines are the VFT (above *T*_g_) and Arrhenius (below *T*_g_) fits of dielectric experimental data. (d) *G*′(*f*) and (e) *G*″(*f*) data of the IL and equimolar mixture with water presented in the form of a master curve. (Inset) Loss modulus *G*″(*f*) spectra of the IL–H_2_O mixture at various temperatures.

From Figure 2c, it is apparent that the glassy dynamics of the IL–H_2_O mixture and dried IL is characterized by two secondary modes β and ν, faster than conductivity relaxation, with the activation energy barrier equal to 20.1 and 51.4 kJ mol^–1^, respectively. While the former stays insensitive to the water content, the latter becomes markedly slower after evaporation of 5.8 wt % H_2_O; however, its activation energy *E*_a_ does not change. Notably, *E*_a_ of the ν mode detected in the examined IL–H_2_O mixture is comparable to activation energy determined for pure water under nanoconfinement.^36^ Consequently, τ_ν_ can be considered to be the accurate fingerprint of water dynamics. Another exciting feature of ν relaxation is its sensitivity to the glass transition of the entire mixture. Specifically, at *T* corresponding well with calorimetric *T*_g_, τ_ν_(*T*^–1^) reveals a characteristic crossover. Note that the temperature dependence of conductivity relaxation times τ_σ_(*T*^–1^) behaves in a similar way, i.e., at *T* > *T*_g_, τ_σ_(*T*^–1^) follows the Vogel–Fulcher–Tamman (VFT) law and becomes Arrhenius below *T*_g_. This behavior is commonly observed in various ionic glass formers.^37−39^ A closer inspection reveals that τ_σ_ at *T*_g_ of dried IL is close to 100 s, the time scale usually identified with the freezing point of ion motions at *T*_g_.^40^ However, τ_σ_(*T*_g_) is 100 times faster for a hydrated sample. This indicates that fast proton hopping contributes to the charge transport of the IL–H_2_O mixture, making the latter much faster than the structural relaxation.^41^ A direct comparison between τ_σ_ and structural dynamics (τ_α_) is needed to confirm such a decoupling. For this purpose, rheological measurements have been performed (see the Supporting Information for details). The selected loss *G*″(*f*) modulus spectra of the IL–H_2_O mixture collected in the vicinity of the liquid–glass transition are shown in the inset of Figure 2e. Because the *G*″(*f*) maxima, defining the structural relaxation time τ_α_ = 1/2π*f*_max_, are directly identified in a limited frequency range of 10^–2^–10^1^ Hz, the time–temperature superposition (TTS) principle was employed to probe the structural relaxation in the supercooled liquid state. The obtained master plots of *G*′(*f*) and *G*″(*f*) data of IL–H_2_O and dried IL collected over a broad temperature range are presented in panels d and e of Figure 2, while τ_α_(*T*^–1^) determined from the shift factor is directly compared to τ_σ_(*T*^–1^) on Figure 2c. As expected, for both examined systems, the time scale of structural rearrangement is longer than that corresponding to charge transport, and for the IL–H_2_O mixture, this difference reaches 3 decades at *T*_g_. Because the decoupling index is markedly smaller for dried IL, one can claim that H_2_O is actively involved in charge transport in the equimolar composition of ions and water molecules. From the master plots presented in Figure 2d, another substantial difference between hydrated and dried IL can be noticed. Specifically, an additional shoulder shows up on the low-frequency side of the *G*′(*f*) spectra of the IL–H_2_O sample. Similar results were reported previously for monohydroxy alcohols (MAs) (e.g., 4-methyl-3-hexanol) and interpreted as a sign of the supramolecular structure.^42^ Thus, one can expect that the formation of water clusters is a source of low-frequency contribution to *G*′(*f*) spectra. To estimate the number of H_2_O molecules forming a cluster (*N*), we employed a simple approach applied before to MAs and polymers.^43^ First, we identified the frequency position of the slowest mode as the kink on the low-frequency side of the scaled modulus (*f* = 3.91 × 10^–4^ Hz; see red arrow in Figure 2d). Next, from the low-frequency limit of *G*″, we determined the steady shear viscosity η_0_ = *G*″/2π*f*. With η_0_ = 1.6 × 10^10^ Pa s, density ρ = 1.406 g/cm^3^ [calculated from the equation of state (EOS) at the same temperature conditions, i.e., 241 K], and previously determined position of slow process *f*, we estimated the effective molecular weight of the cluster . Thus, *N* = *M*_eff_/*M*_H_2_O_ ≈ 6 can be interpreted as the number of molecules confined in the IL. Consequently, one can assume that cations and anions shape water in the cage. The molecular dynamics simulations suggest a similar arrangement of ions and water molecules in a few mixtures containing other imidazolium-based ILs and H_2_O.^44−47^

From this perspective, it is interesting to check whether the dynamics of water clusters are sensitive to substantial density changes and satisfy the scaling concept, ρ^γ^/*T*. For this purpose, we performed isothermal dielectric measurements of the IL–H_2_O mixture at 247 and 258 K. For the representative spectra, collected at various *T*–*P* conditions, however, similar positions of the σ process are shown in Figure 3a. It is evident that both relaxation processes describing the charge transport and the dynamics of water clusters are sensitive to isothermal compression. The analysis of high-pressure spectra with the same protocol as before gives the pressure dependence of τ_σ_ and τ_ν_, displayed in Figure 3b. One can see that τ_σ_ of the supercooled IL–H_2_O mixture becomes longer with the pressure and markedly slows when the liquid–glass transition is achieved. Consequently, an intersection between two linear τ_σ_(*P*) dependences manifests the glass transition pressure (*P*_g_). Note that τ_σ_(*P*_g_) becomes shorter when compression at a higher temperature is performed. This proves that charge transport becomes more efficient under pressure and confirms the contribution of the Grotthuss mechanism to overall charge transport in the examined IL–H_2_O mixture.^48^ From Figure 3b, the pressure evolution of τ_ν_ is also evident; however, in comparison to that of τ_σ_, ν relaxation reveals slightly weaker pressure sensitivity.

**Figure 3:**
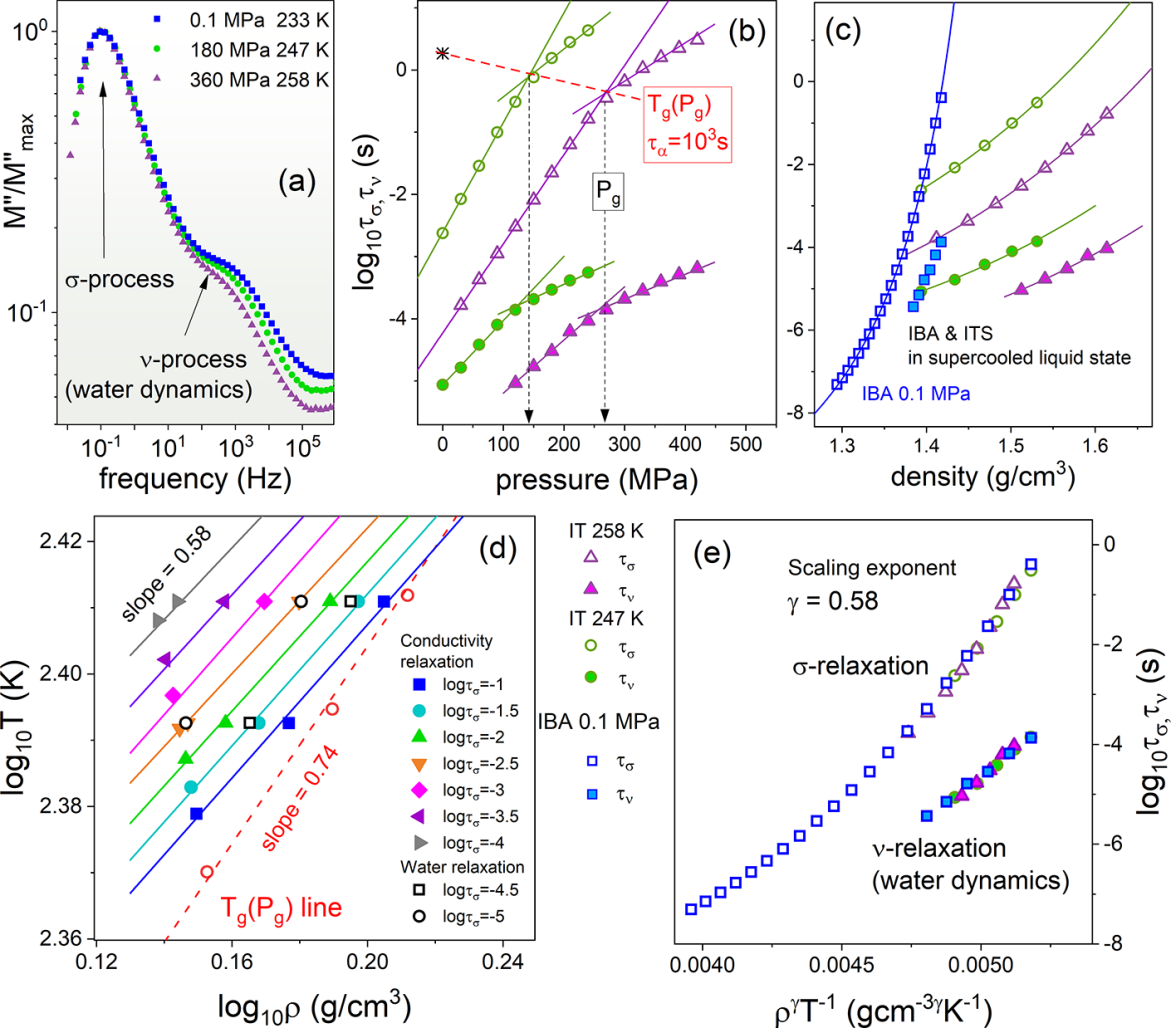
High-pressure data of the IL–H_2_O mixture. (a) Representative *M*″(*f*) peaks recorded at various *T*–*P* conditions. (b) Isothermal pressure dependences upon τ_σ_ and τ_ν_. The crossover of τ_σ_(*P*) denotes the *T*_g_(*P*_g_) line. In panel c, isothemal and isobaric τ_σ_(*P*) and τ_ν_(*P*) data are presented as a function of the density. Panel d presents isochronal log_10_*T* versus log_10_ ρ dependences. Open squares were determined at the isochronal structural relaxation time along the *T*_g_–*P*_g_ line presented in panel b. The γ exponents were determined from the global linear fitting of *T*(ρ) dependences. Panel e presents the scaling curves for conductivity relaxation and water dynamics of the IL–H_2_O mixture.

The next step toward experimental verification of the ρ^γ^/*T* concept is to convert all of the collected τ_ν_(*T*,*P*) and τ_σ_(*T*,*P*) data to density representation. To calculate the specific volume at each *T*–*P* state point, an additional set of *V*_sp_(*T*,*P*) data is required. For this purpose, we performed the *PVT* measurements of the IL–H_2_O mixture and parametrized obtained *V*_sp_(*T*,*P*) dependences by an equation of state (EOS) (see the Supporting Information for more details). The resulting τ_ν_(ρ) and τ_σ_(ρ) dependences collected in the supercooled state are shown in Figure 3c. Note that the relaxation data measured in the glassy phase were omitted because the volume changes accompanying compression were examined only above the liquid–glass transition. On the basis of the obtained isobaric and isothermal log_10_ τ_σ_(ρ) and log_10_ τ_ν_(ρ) data, we have determined a set of isochronal log_10_*T*(log_10_ ρ) dependences (each one at a constant log_10_ τ, i.e., log_10_ τ = −1, −2, ..., −4) that provides a direct estimate of the γ parameter. As presented in Figure 3d, the slope of a double logarithmic plot of *T* versus ρ is practically the same for all isochrones (determined for both σ and ν relaxations) and equal to 0.58 ± 0.02, thereby defining the scaling factor for charge transport (conductivity relaxation) and water dynamics. In turn, a slightly larger γ exponent is obtained from linear regression of log_10_*T*_g_(log_10_ ρ_g_) data (γ^α^ = 0.74; see Figure 3d). This is because the *T*_g_(ρ_g_) line (see Figure 3b) reflects the isochronal conditions for structural relaxation (τ_α_ = 10^3^ s), which was found to be decoupled from conductivity relaxation characterizing the charge transport of the IL–H_2_O mixture. The value of the γ parameter, much lower than unity, suggests that the dynamics of the IL–H_2_O mixture is rather a function of *T*, while the volume plays an almost negligible role. An approach quantifying the relative importance of these two effects is the ratio of the activation energy at constant volume *E*_V_ to the activation enthalpy at constant pressure *E*_p_ easily calculated from relation .^49^ The *E*_V_/*E*_P_ calculated for IL–H_2_O at *T*_g_ and ambient pressure is equal to 0.82, implying a prevailing role of the temperature over the density on the transport properties of this system, as suggested above. Interestingly, the value of *E*_V_/*E*_P_ obtained for IL–H_2_O is higher than those reported before for strongly associated systems (e.g., methanol, *E*_V_/*E*_P_ = 0.58; ethanol, *E*_V_/*E*_P_ = 0.66),^50^ where volume changes do not affect the dynamics much as a result of the strong hydrogen bonding.

Having the parameter γ already determined, one can validate the scaling criterion for water dynamics and conductivity relaxation in the examined IL–H_2_O mixture. As demonstrated in Figure 3e, the isothermal and isobaric dependences of τ_σ_ form a single master curve when plotted as a function of ρ^γ^/*T* with γ = 0.58. Interestingly, ν relaxation reflecting the dynamics of water clusters also satisfies the density scaling with the same exponent. This is in contrast to a breakdown of the thermodynamic scaling observed before for bulk water.^33^ However, the main differences between these two experiments are the explored temperature and pressure ranges. For bulk water, viscosities were determined at high temperatures (*T* = 255 to 363 K) and pressures up to 900 MPa, where the water anomalies are detectable. Furthermore, the number of hydrogen bonds varies substantially over a broad *T*–*P* range, leading to scaling failure in bulk water. At the same time, the hydrogen-bonding network of the examined herein water clusters is somewhat stabilized by surrounding ions. Moreover, the water dynamics has been investigated over a relatively narrow pressure range (up to 300 MPa), the same as reported before for a glycerin strongly hydrogen-bonding system that satisfies the scaling up to 350 MPa, although, over the years, it was considered as a non-scalable glass former.

In summary, this letter presents the experimental ambient and high-pressure studies of a supercooled equimolar aqueous mixture of acidic IL. We found that (i) the conductivity σ relaxation describing the time scale of charge (proton) transport in the IL–H_2_O mixture and (ii) fast secondary ν process, reflecting mobility of water molecules in the cage formed by cations and anions, both obey the density scaling ρ^γ^/*T* rule with the scaling exponent γ equal to 0.58. This confirms that the same short-range intermolecular potential controls both relaxation modes. Furthermore, the obtained γ coefficient, one of the smallest determined thus far, indicates that the dynamics of the IL–H_2_O mixture is a function of *T*, while the role of volume can be almost negligible. Furthermore, we expect that the exponent γ below unity is universal for water dynamics in ion-containing systems. At the same time, the scaling factor characterizing conductivity relaxation depends upon the contribution of proton conductivity to the overall charge transport. Namely, stronger proton conductivity results in a lower γ exponent. From this perspective, the γ factor can be considered to be a new indicator of fast proton hopping.

## Supporting Information Available

Additional experimental details of *PVT*, dielectric, and DSC measurements (PDF)
