# Supplementary material for: Dynamics of Water Clusters Confined in Ionic Liquid at an Elevated Pressure
Source: J Phys Chem Lett. 2024 Mar 18;15(12):3376–82. doi: 10.1021/acs.jpclett.4c00356 (PMC10983063; doi:10.1021/acs.jpclett.4c00356)
Supplement: Supplementary file 1 — jz4c00356_si_001.pdf [file jz4c00356_si_001.pdf]

Supporting Information

Dynamics of Water Clusters Confined in Ionic Liquid at Elevated Pressure

Amith Kumar Murali[1], Marian Paluch[1], R. Casalini[2] Alyna Lange[3], Andreas Taubert[3], Zaneta Wojnarowska[1*]

[1]Institute of Physics, the University of Silesia in Katowice, 75 Pulku Piechoty 1A, 41–500 Chorzow, Poland
[2] Chemistry Division, Naval Research Laboratory, 4555 Overlook Avenue SW, Washington, DC 20375, USA
[3]Institute of Chemistry, University of Potsdam, Karl-Liebknecht-Straße 24-25, 14469 Potsdam-Golm, Germany

Synthesis of [BMIm-SO_3H][pTS].
The ionic liquid was synthesized via a two-step process that is described in detail elsewhere. In short, in the first step, the zwitterion [BMIm-SO_3] is
synthesized by the reaction of 1-methylimidazol and 1,4-butane sultone in acetone. The resulting white powder is then used in the second step to obtain the ionic liquids by mixing the zwitterion with p-toluenesulfonic acid in equimolar amounts.

Experimental methods

Differential Scanning Calorimetry Measurements.

Thermodynamic properties of the studied samples were examined by means of a Mettler-Toledo DSC instrument equipped with a HSS8 ceramic sensor having 120 thermocouples and a liquid nitrogen cooling accessory. The calibrations for temperature and enthalpy were performed by using indium and zinc standards.

Dielectric Measurements.

The dielectric measurements in a wide frequency range of 10^{-2} Hz to 10^{7} Hz were performed at ambient pressure using a Novo-Control GMBH Alpha dielectric spectrometer. The same stainless steel electrodes (diameter = 15 mm) with a fixed distance (0.1 mm) provided by the quartz ring were used for both studied ILs (dried and hydrated). During the measurements, the temperature was controlled by a Novocool system using a nitrogen gas cryostat with an accuracy of 0.1 K.
For the pressure-dependent dielectric measurements, we used the capacitor filled with the studied sample, which was next placed in the high-pressure chamber and compressed using silicone oil. Note that the sample was only in contact with stainless steel during the measurement. The Unipress setup measured the pressure with a resolution of 1 MPa. The temperature was controlled within 0.1 K by means of a Weiss fridge.
The collected dielectric results in the representation of an electric modulus were analyzed by using the Havriliak-Negami (HN) function:

$$M^*(f) = 1/\varepsilon^*(f) = \varepsilon_\infty + \frac{\Delta\varepsilon}{[1+(i2\pi f\tau_{HN})^{\alpha_{HN}}]^{\beta_{HN}}}, \quad (1)$$

where $\Delta\varepsilon$ is the dielectric strength, ε_∞ denotes the high frequency limit permittivity, τ_{HN} is the characteristic relaxation time and the exponents α_{HN} and β_{HN} represent the symmetric and asymmetric broadenings of the dielectric loss curve. For describing the *M"(f)* data in the glassy state the superposition of two Cole-Cole (HN with $\alpha_{HN} \neq 1$ and $\beta_{HN}=1$) functions need to be applied. On the other hand, Cole–Davidson formula (HN with $\alpha_{HN} \neq 1$ and $\beta_{HN}=1$)) was used to parametrrize the experimental data above T_g.

Having the values of fitting parameters, we calculated the characteristic relaxation times of all modes, *α, β* and *ν*:

$$\tau = 1/2\pi f_{\max} = \tau_{HN}\left[\sin\left(\frac{\alpha_{HN}\cdot\pi}{2+2\beta_{HN}}\right)\right]^{-1/\alpha_{HN}}\left[\sin\left(\frac{\alpha_{HN}\cdot\beta_{HN}\cdot\pi}{2+2\beta_{HN}}\right)\right]^{1/\alpha_{HN}} \quad (2)$$

Viscosity measurements.

The mechanical measurements were measured employing an ARES G2 Rheometer. In the supercooled liquid region, aluminum parallel plates of diameter 4 mm were used. The rheological experiments were performed in the frequency range from 0.1 to 100 rad·s^{-1} (10 points per decade) with strain equal to 0.01% in the vicinity of the liquid glass transition.

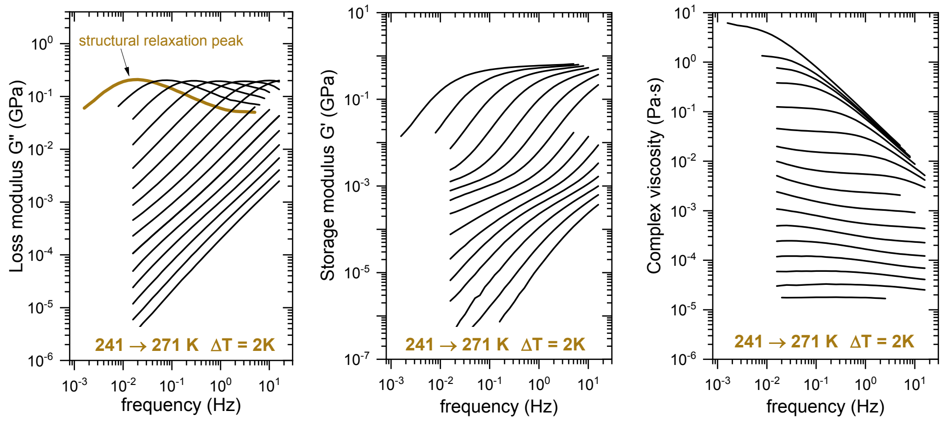

Figure 1S. Results of mechanical measurements of IL-H2O mixture performed in the temperature range 241 K to 271 K.

PVT measurements

These measurements employed a Gnomix dilatometer,[1,2] with mercury as the confining fluid. The pressure range was 10 to 200 MPa, at temperatures from RT to 353 K. The accuracy of the obtained specific volume was 0.002 mL g−1. The Gnomix measures only changes in volume; these were converted to absolute values using the specific volume determined for ambient conditions.

Density measurements at 298 K (1.28 g/cm^3) and ambient conditions were performed by using a vibrating-tube densimeter DMA 4500 M (Anton Paar, Austria). The apparatus was calibrated directly before measurements with dry air and re-distilled water. The water was always freshly degassed (by boiling) before using; its electrolytic conductivity was $1\cdot10^{-4}$ S·m^{-1} at T = 298.15

K. The reference values for air and water were selected based on the formula of Spieweck and Bettin. The instrument was thermostatted within ±0.001 K and viscosity related errors in the density were automatically corrected.

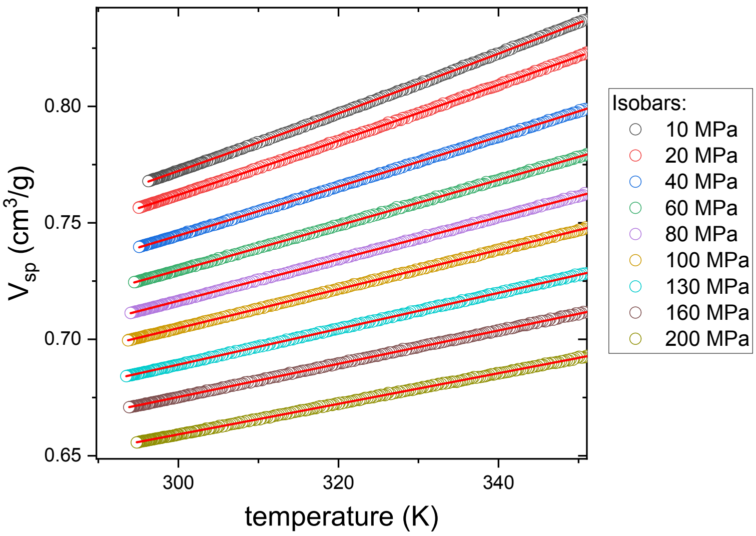

Figure 2S PVT data of IL-H_2O mixture parameterized by Eq. 3

The collected *PVT* data were parameterized by means of an equation of state (Eq.3),[3,4]

$$\left(\frac{v(T,p_0)}{v(T,p)}\right)^{\gamma_{EOS}} = 1 + \frac{\gamma_{EOS}}{B_T(p_0)}(p - p_0) \quad (3)$$

where: $B_T(p_0) = B_{T_0}(p_0)\exp(-b_2(T - T_0))$ and $v(T,p_0) = A_0 + A_1(T - T_0) + A_2(T - T_0)^2$

with the following fitting parameters: A_0= 0.713 cm^3/g**,** A_1=11.2·10^{-4}cm^3/(g·K), A_2=1.40·10^{-6} cm^3/(g·K^2), $B_T(P_0)$= 1010 MPa, b=0.00575 K^{-1}, γ_{EOS}=5.24, T_0= 243 K

[1] Fakhreddin, Y. A.; Zoller, P. ANTEC’91. Society of Plastic Engineers **1991**, *36*, 1642.

[2] Zoller, P.; Walsh, D. Standard Pressure−Volume−Temperature Data for Polymers; Technomic: Lancaster, PA, **1995**.

[3]Grzybowski, A.; Paluch, M.; Grzybowska, K. Consequences of an Equation of State in the Thermodynamic Scaling Regime. *J. Phys. Chem. B* **2009**, *113*, 7419-7422.

[4]Grzybowski, A.; Koperwas, K.; Paluch, M. Equation of State in the Generalized Density Scaling Regime Studied from Ambient to Ultra-High Pressure Conditions. *J. Chem. Phys.* **2014**, *140*, 044502.
